# 2-Amino-5-methyl­pyridinium 6-oxo-1,6-dihydro­pyridine-2-carboxyl­ate

**DOI:** 10.1107/S1600536812041359

**Published:** 2012-10-20

**Authors:** Kaliyaperumal Thanigaimani, Abbas Farhadikoutenaei, Nuridayanti Che Khalib, Suhana Arshad, Ibrahim Abdul Razak

**Affiliations:** a School of Physics, Universiti Sains Malaysia, 11800 USM, Penang, Malaysia

## Abstract

The anion of the title salt, C_6_H_9_N_2_^+^·C_6_H_4_NO_3_^−^, undergoes an enol-to-keto tautomerism during the crystallization. In the crystal structure, the cation and anion are held together by a relatively short N—H⋯O hydrogen bond, and the two anions are further connected to each other by a pair of N—H⋯O hydrogen bonds with an *R*_2_^2^(8) ring motif, thus forming a centrosymmetric 2 + 2 aggregate. The aggregates are further linked through weak N—H⋯O and C—H⋯O hydrogen bonds, resulting a three-dimensional network.

## Related literature

For details of 2-amino­pyridine and its derivatives, see: Banerjee & Murugavel (2004 ▶); Bis & Zaworotko (2005 ▶); Bis *et al.* (2006 ▶). For details of 6-hy­droxy­picolinic acid, see: Sun *et al.* (2004 ▶); Soares-Santos *et al.* (2003 ▶). For a related structure, see: Sawada & Ohashi (1998 ▶). For hydrogen-bond motifs, see: Bernstein *et al.* (1995 ▶). For bond-length data, see: Allen *et al.* (1987 ▶). For stability of the temperature controller used in the data collection, see: Cosier & Glazer (1986 ▶).
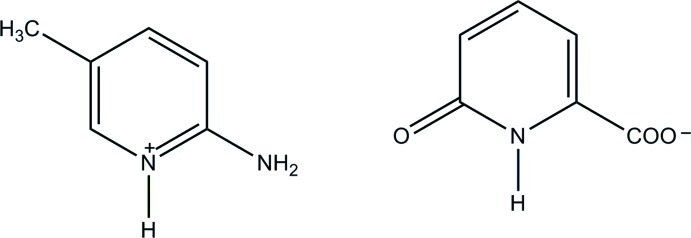


## Experimental

### 

#### Crystal data

C_6_H_9_N_2_^+^·C_6_H_4_NO_3_^−^
*M* *_r_* = 247.25
Monoclinic, 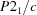

*a* = 11.7093 (6) Å
*b* = 10.4594 (6) Å
*c* = 11.4590 (6) Å
β = 119.203 (1)°
*V* = 1225.03 (11) Å^3^
*Z* = 4
Mo *K*α radiation
μ = 0.10 mm^−1^
*T* = 100 K
0.45 × 0.35 × 0.23 mm

#### Data collection

Bruker SMART APEXII DUO CCD area-detector diffractometer
Absorption correction: multi-scan (*SADABS*; Bruker, 2009 ▶) *T*_min_ = 0.957, *T*_max_ = 0.978
15984 measured reflections
4430 independent reflections
3745 reflections with *I* > 2σ(*I*)
*R*_int_ = 0.027

#### Refinement

*R*[*F* ^2^ > 2σ(*F* ^2^)] = 0.039
*wR*(*F* ^2^) = 0.116
*S* = 1.02
4430 reflections
180 parameters
H atoms treated by a mixture of independent and constrained refinement
Δρ_max_ = 0.43 e Å^−3^
Δρ_min_ = −0.21 e Å^−3^

### 

Data collection: *APEX2* (Bruker, 2009 ▶); cell refinement: *SAINT* (Bruker, 2009 ▶); data reduction: *SAINT*; program(s) used to solve structure: *SHELXTL* (Sheldrick, 2008 ▶); program(s) used to refine structure: *SHELXTL*; molecular graphics: *SHELXTL*; software used to prepare material for publication: *SHELXTL* and *PLATON* (Spek, 2009 ▶).

## Supplementary Material

Crystal structure: contains datablock(s) global, I. DOI: 10.1107/S1600536812041359/is5197sup1.cif

Structure factors: contains datablock(s) I. DOI: 10.1107/S1600536812041359/is5197Isup2.hkl

Supplementary material file. DOI: 10.1107/S1600536812041359/is5197Isup3.cml

Additional supplementary materials:  crystallographic information; 3D view; checkCIF report

## Figures and Tables

**Table 1:** Hydrogen-bond geometry (Å, °)

*D*—H⋯*A*	*D*—H	H⋯*A*	*D*⋯*A*	*D*—H⋯*A*
N1—H1*N*1⋯O1^i^	0.899 (15)	2.011 (15)	2.8922 (10)	166.0 (16)
N3—H2*N*3⋯O1	0.900 (16)	2.245 (19)	3.0373 (12)	146.7 (15)
N3—H2*N*3⋯O3^i^	0.900 (16)	2.408 (16)	3.0916 (11)	133.0 (15)
N3—H1*N*3⋯O2^ii^	0.938 (15)	1.884 (16)	2.8071 (12)	167.7 (15)
N2—H1*N*2⋯O3^i^	0.954 (16)	1.686 (18)	2.6206 (11)	165.7 (17)
C3—H3*A*⋯O1^iii^	0.95	2.33	3.2598 (11)	166
C9—H9*A*⋯O1^iv^	0.95	2.54	3.3750 (12)	146

Symmetry codes: (i) 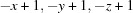; (ii) 
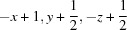
; (iii) 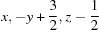; (iv) 
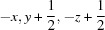
.

**Fig. 1.**
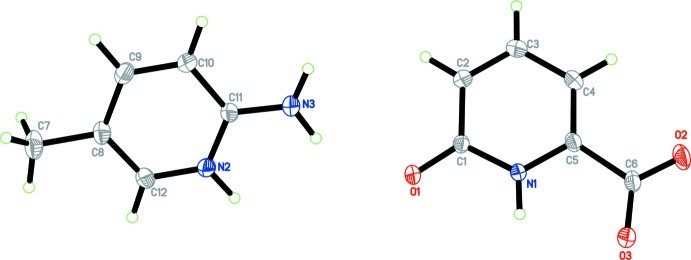
The molecular structure of the title compound with atom labels with 50% probability displacement ellipsoids.

**Fig. 2.**
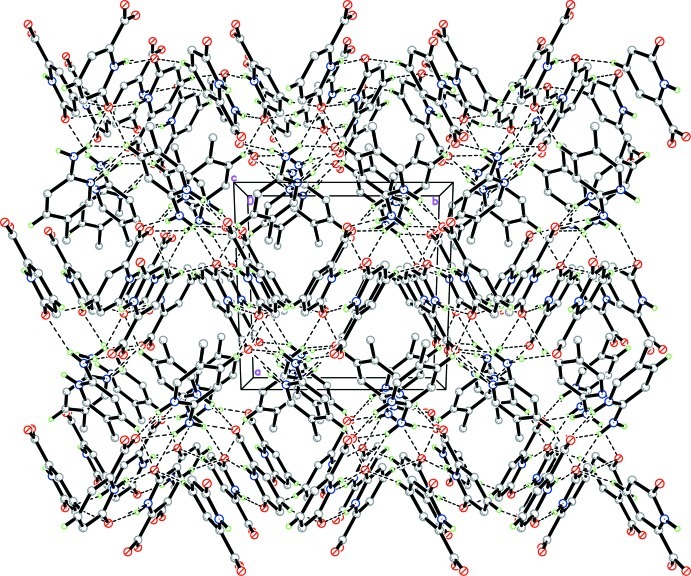
The crystal packing of the title compound, viewed down the c axis.

## supplementary crystallographic information

### Comment

2-Aminopyridine and its derivatives are some of the most frequently used synthons in supramolecular chemistry based on hydrogen bonds (Banerjee & Murugavel, 2004; Bis & Zaworotko, 2005; Bis *et al.*, 2006). 6-Hydroxypicolinic acid has interesting characteristics: firstly, it was characterized by a similar enol-keto tautomerism due to the labile hydrogen atom of –OH group in α-position migrating easily to the basic pyridine N atom; secondly, the multiple coordination sites such as the carbonyl oxygen, the amide nitrogen and carboxylate oxygen atoms are able to coordinate with various metal ions (Sun *et al.*, 2004; Soares-Santos *et al.*, 2003). In order to study some interesting hydrogen bonding interactions of this compound, the synthesis and structure of the title salt is presented here.

The asymmetric unit of the title compound contains a 2-amino-5-methylpyridinium cation and a 6-oxo-1,6-dihydropyridine-2-carboxylate anion (Fig. 1). The 2-amino-5-methylpyridinium cation is planar, with a maximum deviation of 0.004 (1) Å for atoms N2 and C9. In the cation, a wider than normal angle [C11—N2—C12 = 122.96 (8)°] is subtended at the protonated N2 atom. The bond lengths (Allen *et al.*, 1987) and angles are normal. The anion exists in the keto-enol tautomerism of the –CONH moiety. Similar form was also observed in the crystal structure of 2-oxo-1,2-dihydropyridine-6-carboxylic acid (Sawada & Ohashi, 1998).

In the crystal (Fig. 2), the 6-oxo-1,6-dihydropyridine-2-carboxylate anion are centrosymmetrically paired through a pair of N1—H1N1···O1^i^ hydrogen bonds (symmetry code in Table 1) to form an *R*_2_^2^(8) (Bernstein *et al.*, 1995) ring motif. These motifs are further self-organized through N—H···O hydrogen bonds to generate an array of four hydrogen bonds, resulting in the rings with *R*_2_^2^(8), sandwiched by two *R*_2_^2^(7). One of the O atoms of the carboxylate group acts as an acceptors of bifurcated N2—H1N2···O3^i^ and N3—H2N3···O3^i^ hydrogen bonds (symmetry code in Table 1) with the protonated pyridine and amine N atoms of the cation, forming an *R*_2_^1^(6) ring motif. The crystal structure are further stabilized by strong N3—H1N3···O2^ii^ and weak C3—H3A···O1^iii^ and C9—H9A···O1^iv^ hydrogen bonds (symmetry codes in Table 1), resulting a three-dimensional network.

### Experimental

Hot methanol solutions (20 ml) of 2-amino5-methylpyridine (54 mg, Aldrich) and 6-Hydroxypicolinic acid (69 mg, Merck) were mixed and warmed over a heating magnetic stirrer hotplate for a few minutes. The resulting solution was allowed to cool slowly at room temperature and crystals of the title compound (I) appeared after a few days.

### Refinement

N-bound H atoms were located in a difference Fourier map and refined freely [refined N—H distances 0.899 (15), 0.954 (16), 0.900 (16) and 0.938 (15) Å]. The remaining H atoms were positioned geometrically (C—H= 0.95–0.98 Å) and were refined using a riding model, with *U*_iso_(H) = 1.2*U*_eq_(C) or 1.5*U*_eq_(methyl C). A rotating group model was used for the methyl group. In the final refinement, four outliers were omitted (-1 3 2, -4 6 5,-1 1 1 and -4 6 4).

### Crystal data

**Table:**

C_6_H_9_N_2_^+^·C_6_H_4_NO_3_^−^	*F*(000) = 520
*M**_r_* = 247.25	*D*_x_ = 1.341 Mg m^−^^3^
Monoclinic, *P*2_1_/*c*	Mo *K*α radiation, λ = 0.71073 Å
Hall symbol: -P 2ybc	Cell parameters from 6176 reflections
*a* = 11.7093 (6) Å	θ = 3.6–32.6°
*b* = 10.4594 (6) Å	µ = 0.10 mm^−^^1^
*c* = 11.4590 (6) Å	*T* = 100 K
β = 119.203 (1)°	Block, colourless
*V* = 1225.03 (11) Å^3^	0.45 × 0.35 × 0.23 mm
*Z* = 4	

### Data collection

**Table:**

Bruker SMART APEXII DUO CCD area-detector diffractometer	4430 independent reflections
Radiation source: fine-focus sealed tube	3745 reflections with *I* > 2σ(*I*)
Graphite monochromator	*R*_int_ = 0.027
φ and ω scans	θ_max_ = 32.6°, θ_min_ = 3.6°
Absorption correction: multi-scan (*SADABS*; Bruker, 2009)	*h* = −17→17
*T*_min_ = 0.957, *T*_max_ = 0.978	*k* = −15→15
15984 measured reflections	*l* = −16→17

### Refinement

**Table:**

Refinement on *F*^2^	Primary atom site location: structure-invariant direct methods
Least-squares matrix: full	Secondary atom site location: difference Fourier map
*R*[*F*^2^ > 2σ(*F*^2^)] = 0.039	Hydrogen site location: inferred from neighbouring sites
*wR*(*F*^2^) = 0.116	H atoms treated by a mixture of independent and constrained refinement
*S* = 1.02	*w* = 1/[σ^2^(*F*_o_^2^) + (0.0661*P*)^2^ + 0.2292*P*] where *P* = (*F*_o_^2^ + 2*F*_c_^2^)/3
4430 reflections	(Δ/σ)_max_ < 0.001
180 parameters	Δρ_max_ = 0.43 e Å^−^^3^
0 restraints	Δρ_min_ = −0.21 e Å^−^^3^

### Special details

**Table:**

Experimental. The crystal was placed in the cold stream of an Oxford Cryosystems Cobra open-flow nitrogen cryostat (Cosier & Glazer, 1986) operating at 100.0 (1) K.
Geometry. All e.s.d.'s (except the e.s.d. in the dihedral angle between two l.s. planes) are estimated using the full covariance matrix. The cell e.s.d.'s are taken into account individually in the estimation of e.s.d.'s in distances, angles and torsion angles; correlations between e.s.d.'s in cell parameters are only used when they are defined by crystal symmetry. An approximate (isotropic) treatment of cell e.s.d.'s is used for estimating e.s.d.'s involving l.s. planes.
Refinement. Refinement of *F*^2^ against ALL reflections. The weighted *R*-factor *wR* and goodness of fit *S* are based on *F*^2^, conventional *R*-factors *R* are based on *F*, with *F* set to zero for negative *F*^2^. The threshold expression of *F*^2^ > σ(*F*^2^) is used only for calculating *R*-factors(gt) *etc*. and is not relevant to the choice of reflections for refinement. *R*-factors based on *F*^2^ are statistically about twice as large as those based on *F*, and *R*- factors based on ALL data will be even larger.

### Fractional atomic coordinates and isotropic or equivalent isotropic displacement parameters (Å^2^)

**Table:**

	*x*	*y*	*z*	*U*_iso_*/*U*_eq_	
O1	0.37734 (6)	0.60282 (6)	0.39711 (6)	0.01806 (13)	
O2	0.80810 (7)	0.48126 (7)	0.29773 (7)	0.02633 (15)	
O3	0.79587 (6)	0.45930 (6)	0.48584 (7)	0.02282 (14)	
N1	0.55569 (6)	0.55843 (7)	0.37336 (7)	0.01512 (13)	
N2	0.02845 (7)	0.72276 (7)	0.43791 (7)	0.01818 (14)	
N3	0.14291 (7)	0.77377 (8)	0.32695 (8)	0.02345 (16)	
C1	0.43630 (7)	0.61584 (7)	0.33157 (8)	0.01518 (14)	
C2	0.38707 (8)	0.68852 (8)	0.21019 (8)	0.02096 (16)	
H2A	0.3064	0.7328	0.1776	0.025*	
C3	0.45395 (9)	0.69519 (10)	0.14089 (9)	0.02493 (18)	
H3A	0.4195	0.7439	0.0608	0.030*	
C4	0.57443 (9)	0.63000 (9)	0.18740 (9)	0.02318 (17)	
H4A	0.6203	0.6326	0.1382	0.028*	
C5	0.62317 (8)	0.56351 (8)	0.30428 (8)	0.01706 (15)	
C6	0.75338 (8)	0.49530 (8)	0.36664 (9)	0.01855 (15)	
C7	−0.25015 (10)	0.86990 (10)	0.46610 (12)	0.0311 (2)	
H7A	−0.2490	0.7998	0.5234	0.047*	
H7B	−0.3361	0.8737	0.3856	0.047*	
H7C	−0.2328	0.9509	0.5149	0.047*	
C8	−0.14653 (8)	0.84727 (8)	0.42693 (9)	0.02107 (16)	
C9	−0.12821 (9)	0.93128 (9)	0.34046 (10)	0.02358 (17)	
H9A	−0.1834	1.0039	0.3055	0.028*	
C10	−0.03306 (9)	0.91053 (9)	0.30608 (9)	0.02217 (17)	
H10A	−0.0219	0.9685	0.2486	0.027*	
C11	0.04861 (7)	0.80162 (8)	0.35711 (8)	0.01760 (15)	
C12	−0.06528 (8)	0.74369 (8)	0.47303 (8)	0.01970 (16)	
H12A	−0.0746	0.6849	0.5310	0.024*	
H1N1	0.5904 (14)	0.5129 (15)	0.4496 (14)	0.033 (3)*	
H2N3	0.1961 (15)	0.7063 (15)	0.3644 (15)	0.037 (4)*	
H1N3	0.1663 (14)	0.8348 (15)	0.2824 (14)	0.034 (3)*	
H1N2	0.0830 (15)	0.6489 (15)	0.4686 (16)	0.040 (4)*	

### Atomic displacement parameters (Å^2^)

**Table:**

	*U*^11^	*U*^22^	*U*^33^	*U*^12^	*U*^13^	*U*^23^
O1	0.0171 (3)	0.0218 (3)	0.0192 (3)	0.0032 (2)	0.0119 (2)	0.0018 (2)
O2	0.0276 (3)	0.0276 (3)	0.0359 (4)	0.0033 (2)	0.0250 (3)	0.0001 (3)
O3	0.0197 (3)	0.0248 (3)	0.0278 (3)	0.0060 (2)	0.0146 (2)	0.0074 (2)
N1	0.0151 (3)	0.0164 (3)	0.0162 (3)	0.0019 (2)	0.0095 (2)	0.0018 (2)
N2	0.0169 (3)	0.0183 (3)	0.0205 (3)	0.0015 (2)	0.0101 (2)	0.0027 (2)
N3	0.0218 (3)	0.0263 (4)	0.0279 (4)	0.0065 (3)	0.0166 (3)	0.0067 (3)
C1	0.0146 (3)	0.0155 (3)	0.0166 (3)	0.0011 (2)	0.0084 (3)	−0.0002 (2)
C2	0.0188 (3)	0.0228 (4)	0.0218 (4)	0.0046 (3)	0.0104 (3)	0.0068 (3)
C3	0.0245 (4)	0.0297 (4)	0.0229 (4)	0.0045 (3)	0.0134 (3)	0.0097 (3)
C4	0.0245 (4)	0.0278 (4)	0.0234 (4)	0.0029 (3)	0.0165 (3)	0.0062 (3)
C5	0.0178 (3)	0.0177 (3)	0.0201 (3)	0.0008 (2)	0.0128 (3)	0.0003 (3)
C6	0.0184 (3)	0.0156 (3)	0.0269 (4)	0.0005 (2)	0.0152 (3)	0.0003 (3)
C7	0.0284 (4)	0.0308 (5)	0.0453 (6)	−0.0001 (4)	0.0269 (4)	−0.0044 (4)
C8	0.0190 (3)	0.0219 (4)	0.0259 (4)	−0.0008 (3)	0.0138 (3)	−0.0025 (3)
C9	0.0213 (4)	0.0218 (4)	0.0293 (4)	0.0058 (3)	0.0137 (3)	0.0036 (3)
C10	0.0223 (4)	0.0217 (4)	0.0246 (4)	0.0053 (3)	0.0131 (3)	0.0063 (3)
C11	0.0160 (3)	0.0194 (3)	0.0182 (3)	0.0009 (2)	0.0090 (3)	0.0009 (3)
C12	0.0195 (3)	0.0210 (4)	0.0215 (4)	−0.0020 (3)	0.0122 (3)	−0.0002 (3)

### Geometric parameters (Å, º)

**Table:**

O1—C1	1.2511 (9)	C3—H3A	0.9500
O2—C6	1.2447 (9)	C4—C5	1.3628 (12)
O3—C6	1.2617 (11)	C4—H4A	0.9500
N1—C5	1.3654 (9)	C5—C6	1.5104 (11)
N1—C1	1.3751 (10)	C7—C8	1.5036 (12)
N1—H1N1	0.899 (15)	C7—H7A	0.9800
N2—C11	1.3440 (11)	C7—H7B	0.9800
N2—C12	1.3585 (10)	C7—H7C	0.9800
N2—H1N2	0.954 (16)	C8—C12	1.3665 (12)
N3—C11	1.3402 (10)	C8—C9	1.4169 (13)
N3—H2N3	0.900 (16)	C9—C10	1.3681 (12)
N3—H1N3	0.938 (15)	C9—H9A	0.9500
C1—C2	1.4361 (11)	C10—C11	1.4172 (11)
C2—C3	1.3622 (12)	C10—H10A	0.9500
C2—H2A	0.9500	C12—H12A	0.9500
C3—C4	1.4161 (12)		
			
C5—N1—C1	123.96 (7)	O2—C6—O3	126.76 (8)
C5—N1—H1N1	118.0 (9)	O2—C6—C5	117.96 (8)
C1—N1—H1N1	118.0 (9)	O3—C6—C5	115.27 (7)
C11—N2—C12	122.96 (7)	C8—C7—H7A	109.5
C11—N2—H1N2	116.1 (9)	C8—C7—H7B	109.5
C12—N2—H1N2	121.0 (9)	H7A—C7—H7B	109.5
C11—N3—H2N3	120.9 (9)	C8—C7—H7C	109.5
C11—N3—H1N3	119.2 (9)	H7A—C7—H7C	109.5
H2N3—N3—H1N3	118.4 (13)	H7B—C7—H7C	109.5
O1—C1—N1	120.55 (7)	C12—C8—C9	116.59 (8)
O1—C1—C2	124.11 (7)	C12—C8—C7	121.36 (8)
N1—C1—C2	115.33 (7)	C9—C8—C7	122.05 (8)
C3—C2—C1	121.23 (7)	C10—C9—C8	121.70 (8)
C3—C2—H2A	119.4	C10—C9—H9A	119.2
C1—C2—H2A	119.4	C8—C9—H9A	119.2
C2—C3—C4	120.43 (8)	C9—C10—C11	119.28 (8)
C2—C3—H3A	119.8	C9—C10—H10A	120.4
C4—C3—H3A	119.8	C11—C10—H10A	120.4
C5—C4—C3	118.57 (7)	N3—C11—N2	119.21 (8)
C5—C4—H4A	120.7	N3—C11—C10	122.91 (8)
C3—C4—H4A	120.7	N2—C11—C10	117.88 (7)
C4—C5—N1	120.42 (7)	N2—C12—C8	121.60 (8)
C4—C5—C6	123.23 (7)	N2—C12—H12A	119.2
N1—C5—C6	116.33 (7)	C8—C12—H12A	119.2
			
C5—N1—C1—O1	176.99 (7)	C4—C5—C6—O3	167.03 (9)
C5—N1—C1—C2	−2.64 (11)	N1—C5—C6—O3	−11.36 (11)
O1—C1—C2—C3	−177.60 (9)	C12—C8—C9—C10	−0.71 (14)
N1—C1—C2—C3	2.02 (12)	C7—C8—C9—C10	179.51 (9)
C1—C2—C3—C4	0.03 (15)	C8—C9—C10—C11	0.60 (14)
C2—C3—C4—C5	−1.64 (15)	C12—N2—C11—N3	−179.75 (8)
C3—C4—C5—N1	1.11 (14)	C12—N2—C11—C10	−0.56 (12)
C3—C4—C5—C6	−177.22 (8)	C9—C10—C11—N3	179.19 (9)
C1—N1—C5—C4	1.13 (13)	C9—C10—C11—N2	0.03 (13)
C1—N1—C5—C6	179.56 (7)	C11—N2—C12—C8	0.45 (13)
C4—C5—C6—O2	−12.31 (13)	C9—C8—C12—N2	0.19 (13)
N1—C5—C6—O2	169.31 (8)	C7—C8—C12—N2	179.97 (8)

### Hydrogen-bond geometry (Å, º)

**Table:**

*D*—H···*A*	*D*—H	H···*A*	*D*···*A*	*D*—H···*A*
N1—H1*N*1···O1^i^	0.899 (15)	2.011 (15)	2.8922 (10)	166.0 (16)
N3—H2*N*3···O1	0.900 (16)	2.245 (19)	3.0373 (12)	146.7 (15)
N3—H2*N*3···O3^i^	0.900 (16)	2.408 (16)	3.0916 (11)	133.0 (15)
N3—H1*N*3···O2^ii^	0.938 (15)	1.884 (16)	2.8071 (12)	167.7 (15)
N2—H1*N*2···O3^i^	0.954 (16)	1.686 (18)	2.6206 (11)	165.7 (17)
C3—H3*A*···O1^iii^	0.95	2.33	3.2598 (11)	166
C9—H9*A*···O1^iv^	0.95	2.54	3.3750 (12)	146

Symmetry codes: (i) −*x*+1, −*y*+1, −*z*+1; (ii) −*x*+1, *y*+1/2, −*z*+1/2; (iii) *x*, −*y*+3/2, *z*−1/2; (iv) −*x*, *y*+1/2, −*z*+1/2.
